# Extracellular microRNAs: key players to explore the outcomes of in vitro fertilization

**DOI:** 10.1186/s12958-021-00754-9

**Published:** 2021-05-15

**Authors:** Haroon Latif Khan, Shahzad Bhatti, Sana Abbas, Celal Kaloglu, Ahmed M. Isa, Hooria Younas, Rachel Ziders, Yousaf Latif Khan, Zahira Hassan, Bilgün Oztürk Turhan, Aysegul Yildiz, Hikmet Hakan Aydin, Ender Yalcinkaya Kalyan

**Affiliations:** 1 Lahore Institute of Fertility and Endocrinology, Hameed Latif Hospital, 14 Abu-Bakar Block New Garden Town, 54800 Lahore, Pakistan; 2 Department of Human Genetics and Molecular biology, University of Health Sciences, Lahore, 54600 Pakistan; 3 Department of Medical Education, Rashid Latif Medical College, Lahore, Pakistan; 4 Department of Histology and Embryology, Cumhuriyet University Faculty of Medicine, 58140 Sivas, Turkey; 5 Assisted Conception Unit, Obstetrics and Gynecology Department, College of Medicine, King Saud University, Riyadh, Saudi Arabia; 6 Department of Biochemistry, Kinnaird College Lahore, Lahore, Pakistan; 7 Your Family Fertility, 1408 Sweet Home Road Suite 9, Amherst, NY 14228 USA; 8 Department of Cellular Pathology, Royal Free Hospital, London, NW3 2QG UK; 9 Department of Obstetrics and Gynecology, Nisdetiye Maha, İstanbul, Turkey; 10 Department of Molecular Biology and Genetics, Faculty of Science, Mugla Sitki Kocman University, Kotekli, 48000 Mugla, Turkey; 11 Department of Medical Biochemistry, Ege University School of Medicine, Bornova, Izmir, Turkey; 12 Department of IVF unit, Private Adatip Hospital, Yenisehir mahallesi Kardelen sokak 2, Pendik, 34912 Istanbul, Turkey

**Keywords:** PCOS, miRNA expression, Follicular fluid, IVF, Embryo quality

## Abstract

**Background:**

MicroRNAs (miRNAs) are small RNA molecules that modulate post-transcriptional gene regulation. They are often used as promising non-invasive biomarkers for the early diagnosis of cancer. However, their roles in assisted reproduction are still unknown.

**Methods:**

This prospective study was designed to evaluate the expression profiles of seven extracellular miRNAs (miR-7-5p, miR-202-5p, miR-378-3p, miR-224, miR-320a, miR-212-3p, and miR-21-5p) in human follicular fluid (FF) to explore the outcomes of in vitro fertilization (IVF). Of 255 women, 145 were without polycystic ovary syndrome (PCOS), and their ovarian assets were normal (NOR), while 110 were with normo-androgenic PCOS.

**Results:**

The combination of six FF miRNAs expression profile discriminated between PCOS and NOR women with a sensitivity of 79.2% and a specificity of 87.32% (AUC = 0.881 [0.61; 0.92], *p* = 0.001). MiR-202-5p significantly had a lower abundance level, and miR-378-3p had a high abundance level in pooled FF samples from patients treated with human menopausal gonadotropin (hMG) than those treated with recombinant follicle-stimulating hormone (rFSH) (*p* < 0.001). Our results showed that miRNA-320a was significantly different in top-quality embryos versus non-top-quality embryos on day 3 in NOR patients with a sensitivity of 80% and specificity of 71%, (AUC = [0.753 (0.651; 0.855)], *p* = 0.001). For clinical pregnancy outcome prediction, FF miRNA-21 exhibited high sensitivity (74.8%) and specificity (83.7%) with the AUC value of 0.774 (0.682; 0.865).

**Conclusion:**

Conclusively, our results provide evidence that miR-7-5p, miR-378-3p, miR-224, miR-212-3p were a differentially high expression in normo-androgenic PCOS patients than NOR patients. While miRNA-320a was significantly different in top-quality embryos versus non-top-quality embryos on day 3 (*p* = 0.001). The expression level of FF miR-212-3p was significantly related to the probability of embryos to develop into a high-quality blastocyst in patients with normal ovarian reserve.

**Supplementary Information:**

The online version contains supplementary material available at 10.1186/s12958-021-00754-9.

## Background

Extracellular microRNAs (miRNAs) are among the most abundant yet enigmatic biomolecules. Belonging to a large group of endogenously expressed single-stranded RNA molecules, ranging from 21 to 24 nucleotides in length, they regulate a wide array of biological processes. They do this by silencing genes and typically work as transcriptional and post-transcriptional regulators of gene expression. MiRNAs do not encode proteins themselves but possess specific binding sites to messenger RNA (mRNA) targets, promoting their decay and inhibiting translation [1]. In mammalian reproduction, miRNAs are markedly involved in controlling several reproductive functions in females, such as folliculogenesis, steroidogenesis, oocyte maturation, early embryonic development, cell proliferation, and apoptosis [2]. They are primarily expressed in all human reproductive tissues and biological fluids, including follicular fluid (FF) [3]. Extracellular miRNAs can be readily quantified as they are highly conserved and notably stable in a harsh environment with the presence of endogenous RNase activity [4]. Seemingly, they are often used as promising non-invasive biomarkers for the early diagnosis of cancer; however, their roles in assisted reproduction treatments remain unknown [5].

Currently, the ovarian reserve assessment is inferred through serum levels of anti-Mullerian hormone (AMH) and basal antral follicle count (AFC) by consultants. These markers are also routinely used to monitor the ovarian response during gonadotrophin therapy. Nonetheless, it became increasingly distinct that variability between laboratory procedures and lack of a standardized international assay is one of the fundamental limitations of AMH testing in ART [6]. Polycystic ovary syndrome (PCOS) is the most frequent endocrine disorder, which has an intricate inheritance pattern among multifaceted endocrinopathies, affecting women of childbearing age. Abnormal preantral folliculogenesis in PCOS is the principal cause of more than 75% of cases of anovulatory infertility and menstrual cycle disturbances [7].

Altered miRNAs expression profile has been reported in the serum and FF of patients with PCOS. Therefore, these differential miRNAs might be classified as specific therapeutic targets and diagnostic biomarkers of PCOS. They could also reflect the real status of fertilization potential in couples undergoing IVF/intracytoplasmic sperm injection (ICSI) treatment [8].

Over the past decade, numerous articles have explained that several miRNAs are fundamentally concerned with the steroidogenic regulation of ovarian follicles, yet the impact of gonadotropin therapy on miRNA expression in the follicular microenvironment remains within the exploratory phase [9, 10]. Specifically, human FF is a complex biological fluid with intrinsic regulation of the micro-environment, determining the oocyte developmental potential and post-fertilization development of an embryo. Subsequently, metabolic changes in its components have been designated as ideal noninvasive biomarkers to predict oocyte and embryo quality. Additionally, by comparing gene expression profiles, it was revealed that miRNAs might be key mediators in specific disease etiology and certain cellular processes; notably, they have an immense contribution as a regulator of oogenesis and embryogenesis [11, 12].

Circulating miRNA-21 acts as key oncomiRs, which are extensively overexpressed in numerous cancer forms, and multiple studies have demonstrated that aberrant overexpression endorses proliferation, invasion, and migration through downregulation of various tumor suppressor genes [13, 14]. However, the downregulation of miRNA-21-5p in granulosa cells (GCs) promotes apoptosis by targeting PI3K/AKT and JAK/STAT3 kinase cascades [15]. It is also linked with RNA-induced silencing complex in GCs and regulates cell proliferation, differentiation, and survival through TGF-β signaling [16, 17]. Earlier studies exhibited that miR-320a is of the top ten highly expressed miRNAs identified in the FF samples and positively linked with embryo quality [18, 19]. Several studies have established that miR-7-5p acts as a key mediator in the feedback loop of the hypothalamus-pituitary-ovary axis by regulating gonadotropins, and knockout leads to infertility due to hypogonadism in the mouse model [20–22]. Previous evidence from in vitro study demonstrated that miR-212 has a potential role in regulating gonadotropins, including follicular development and ovulation in equines [23]. The miR-202-5p is the most dominant form of the miRNAs abundantly expressed in GCs and non-fertilized oocytes. The knockout of miR-202-5p compromises the quantity and quality of oocytes [24]. Likewise, the significant upregulation of miR-378-3p increases the density of the primordial follicles in the ovary. It also induces an increased autophagy level in response to oxidative stress and delays the apoptotic events inside the developing follicles [25]. Li and his fellows highlight the diverse role of miR-224 in ovaries and a promising marker of PCOS. They explained that the overexpression of miR-224 reduces the Ptx3 and Smad4 gene expression responsible for cell expansion and oocyte development [26].

This contemporary study was designed with a primary objective to seek out seven extracellular miRNAs (7-5p, miR-202-5p, miR-378-3p, miR-224, miR-320a, miR-212-3p, miR-21-5p) expression profiles in the follicular microenvironment of IVF patients with polycystic ovaries and with normal ovaries. Further, we examined the importance of these miRNAs to predict the success of ARTs in normal responders.

## Methods

### Subjects

This prospective study was conducted on 255 women undergoing IVF/ICSI at the Lahore Institute of Fertility and Endocrinology, Hameed Latif Hospital Lahore, between January 2017 to January 2020. Among 255 women, 145 with normal ovarian reserve (NOR), while 110 women had normo-androgenic PCOS, diagnosed based on Rotterdam criteria, i.e., clinical or biochemical hyperandrogenism, oligo-and/or anovulation, and appearance of polycystic ovaries on ultrasound examination. The functional ovarian response was determined by measuring the AMH concentration in serum and AFC through transvaginal sonography on cycle day 3. Patients were informed about the study’s objective, and written informed consent was taken before collecting FF samples. This study was approved by the institutional ethical review board, and research was conducted under the recommended guidelines.

### IVF procedure

Of 255 women, 141 received gonadotrophin-releasing hormone (GnRH) agonist (Decapeptyl, Ipsen Pharma), and 114 were undergoing COS (Controlled ovarian stimulation) with antagonist protocol. Two types of gonadotropins were used in COS: r-FSH and HP-hMG. Serum E2 levels and folliculograms were used to assess the ovarian response. Ovulation was triggered with exogenous human chorionic gonadotropin (hCG) (LG, Life Sciences 5000–10,000 IU) once 3 follicles reached a mean diameter of ≥18 mm. After 36 h of hCG administration, oocyte retrieval was performed utilizing transvaginal ultrasound and a single lumen needle (Cook, KOPS-1230-VUB, Limerick, Ireland). Flushing during these retrievals was prevented, and cumulus-oocyte complexes (COC) were removed mechanically. FF samples were stored at − 80 °C for further evaluation. Oocytes were cultured singly in 30 μl of cleavage medium (Vitrolife) and under mineral oil (Ovoil™) at 37 °C in a humid setting containing O_2_ 5%, CO_2_ 6%, and N_2_ 89%, respectively. The fertilization examination was done after 18 h of insemination, and normal fertilization is demonstrated by the appearance of two pronuclei (2PN) and two polar bodies.

Embryo quality was documented on day 3 based on the cleavage-stage-scoring system, including the number of cells, blastomere regularity, degree of fragmentation, and multinucleation. Top-quality embryos on day 3 were considered those with 6–8 regular blastomeres and less than 15% fragmentation [27]. Blastocysts were vitrified and kept in liquid nitrogen (− 196 °C) until embryo transfer. Morphological evaluation of blastocysts was done through the Gardner scoring system [28], and only fully expended blastocysts (IV/V grade) with cohesive trophectoderm (A/B quality) were warmed in a closed vitrification system and transferred under the procedure recommended by Irvine Scientific. Clinical pregnancy was verified by fetal cardiac activity with at least one gestational sac detected on ultrasonography.

### Follicular fluid (FF) sample preparation

After egg retrieval, intrafollicular fluid from both groups (110 PCOS and 145 NOR) pooled separately and subject to centrifugation at 4000 rpm for 15 min. However, in NOR patients, 0.5 mL of the FF from each mature follicle (*n* = 280) with ≥18 mm diameter having a volume of 2–3 cm^3^ were handled separately to associate the miRNAs profiles with embryo quality or with pregnancy rate. Moreover, in NOR patients, each mature oocyte, related embryo, and FF sample were processed separately in the IVF laboratory. Clear supernatant fluids were separated and filtered, applying 0.45 μL syringe filters into sterile 10 ml tubes by excluding dead cells and then stored at − 80 °C. All follicular aspirations were performed between 8: 30 h and 10.00 h.

### Hormonal analysis

BMI was calculated based on height and weight through standardized equipment. Venous blood was drawn between 8: 00 am to 9: 00 am. Baseline hormones such as follicular stimulating hormone (FSH), luteinizing hormone (LH), 17β-estradiol (E_2_), thyroid-stimulating hormone (TSH), Total Testosterone (TT), sex hormone-binding globulin (SHBG), and anti-mullerian duct hormone (AMH) were assessed on 2nd day of the menstrual cycle through electrochemiluminescence immunoassay, according to the manufacturer’s instructions (Elecsys® Roche Diagnostics, Indianapolis, USA). The antral follicle count (AFC) was assessed using transvaginal ultrasonography (TVS) on the 2nd or 3rd day of the menstrual cycle.

### MicroRNAs (miRNA) extraction

MiRNAs extraction was done according to the manufacturer’s protocol. Specific miRNAs were evaluated from 4 ml of each FF pool using QIAamp Circulating Nucleic Acid Kit (Qiagen). In short, in the lysis step, 3 ml of FF sample pool with 500 μL Proteinase K (20 mg/ml) and 4.5 μL of Buffer ACL were mixed that inactivated the DNases and RNases, resulting in the release of nucleic acids from vesicles. Then the mixture was incubated at 60 °C for 30 min. Next, to improve miRNAs’ binding capability to the membrane, 10 ml ABC buffer was added to the lysate and shifted lysate onto a QIAamp Mini Column after mixing by pulse vertexing. The miRNAs were incorporated onto a small silica membrane as the mixture is drawn through by vacuum pressure. The residual contaminants were thoroughly washed away after three steps-washing of the membrane, and pure circulating nucleic acids were eluted in buffer AVE at room temperature.

### Evaluation of miRNAs differential expression profile of human FF through qRT-PCR

The candidate miRNAs assay was determined through qRT-PCR with TaqMan™ MicroRNA assay (Applied Biosystems), and universal stem-loop primers (USLP) with a low intra-assay coefficient of variation (ICV) were used to generate complementary DNA (cDNA). The 15 μL of RT reaction mix comprised FF pool sample: 5 μL (1–10 ng of total RNA), 100 mM dNTP: 0.15 μL, 10X RT buffer: 1.5 μL, 50 U/ μL MultiScribe™ RT enzyme: 1 μL, 20 U/ μL RNase inhibitors: 0.19 μL, nuclease-free water: 4.16 μL, and TaqMan™ 5X RT primers: 3 μL. The reverse transcription was performed under standard cycling conditions: 16 °C for 30 min, 42 °C for 30 min, 85 °C for 5 min, and hold step at 4 °C. CFX-96® touch RT-PCR detection system (Bio-Rad-Life-Sciences, USA) was used for quantitative PCR, carried out in a 384-well plate, and duplicated for each sample with negative control (water).

The 10 μL PCR reaction mix contained cDNA: 3 μL, PCR MasterMix: 5 μL (TaqMan®, Applied Biosystems), and primer: 2 μL under the following conditions: initial denaturation step at 95 °C for 10 min, 50 cycles of 95 °C for 15 s, and anneal at 60 °C for 60 s. miRNA-16 was applied as endogenous control [29], and eq. 2-∆Ct was used to determine the relative expression of miR-7-5p, miR-202-5p, miR-378-3p, miR-224, miR-320a, miRNA-212-3p, miR-21-5p in each FF pool. These seven miRNAs were selected based on their extensive clinical importance in oocyte maturation and embryo quality [11, 19, 30, 31]. We estimated the fold change by evaluating the relative expression levels between normo-androgenic PCOS patients and NOR patients by applying the 2^-∆∆Crt^ method and normalized to miR-16 expression in FF [32, 33].

### Pathway analysis and gene ontology (Go)

MiRNAs were uploaded for KEGG pathway enrichment analysis and Gene Ontology (GO). Putative target genes of studied miRNAs were predicted through eight available bioinformatic algorithms such as miRanda (www.cbio.mskcc.org/miRNA2003/miranda.html) version 1.9, TargetScan (http://www.targetscan.org/vert_72/) version 7.2, DIANA-microT (http://diana.imis.athena-innovation.gr/DianaTools/index.php?r=microT_CDS/index) version 4, miRDB (http://www.mirdb.org/), PicTar (https://pictar.mdc-berlin.de/), miRWalk (http://zmf.umm.uni-heidelberg.de/apps/zmf/mirwalk2/), RNA hybrid (https://bibiserv.cebitec.uni-bielefeld.de/rnahybrid/), and target gene prediction at EMBLE (http://www.russell.embl.de/miRNAs/). However, to minimize the number of false positives targets, we include only those target genes that were predicted by 6 out of 8 programs and proceeded for further use in GO annotation and KEGG (keto Encyclopedia of Genes and Genomes) pathway enrichment analysis using DAVID (https://david.ncifcrf.gov/) version 6.8 integrate functional genomic annotation [34]. Benjamini and Hochberg’s multiple testing correlation was used to adjust the *p*-values (−log 10 FDR adjusted *p* values = < 0.05).

### Statistical analysis

Categorical variables are presented as numbers and percentages, and continuous parametric data are shown as mean ± standard deviation (SD). The Shapiro-Wilk test was used to verify the normality of data, while to compare and correlate quantitative variables, we employed the Mann-Whitney and the Spearman Rank tests, respectively. Univariate analysis was conducted to relate the relationship with PCOS for clinical parameters and each FF miRNA relative expression. Those miRNAs whose relative expressions were significantly associated with the PCOs (*p* < 0.05) in univariate analysis were further subjected to multivariate analysis. Similarly, univariate analysis showed that BMI trajectory had significantly associated with PCOS, thus integrated into the multivariate analysis to calculate the adjusted odds ratios for the six miRNAs. ROC curves were plotted to find the cutoff values regarding FF miRNAs’ ability to discriminate between normo-androgenic PCOs and normal ovarian reserves. We also predicted the blastocyst developmental potential and clinical pregnancy outcomes by calculating the area under the curve [AUC (95% CI)]. Sensitivity and specificity were calculated for the optimal cutoff values using XLSTAT 2020 software and SPSS (version 27; SPSS Inc., Chicago, IL.USA). *P*-values less than 0.05 were considered statistically significant.

## Results

### Baseline clinical characteristics of the subjects

The baseline clinical characteristics of all subjects (110 PCOS and 145 NOR women) were given in Table 1. A significant difference was perceived in terms of FSH, LH, AMH, LH/FSH ratio, AFC, and total dose of gonadotrophins between women with normo-androgenic PCOS compared with those possessing normal ovarian reserve (NOR) (*p* < 0.05). However, age, BMI, duration of infertility, estradiol levels, total testosterone, prolactin, TSH, and SHBG concentrations were not significantly different between normo-androgenic PCOS and NOR patients. Furthermore, the number of retrieved oocytes, the number of MII- oocytes, rate of mature oocytes, fertilization rate, and total fragmentation rate were significantly higher in PCOS than in the NOR group (*p* < 0.05). The blastulation rate, expanded blastocyst rate, and clinical pregnancy rate per transfer was significantly increased in the NOR group compared to the PCOS group (*p* < 0.05), as presented in Table 2.

**Table 1:** Baseline clinical characteristics of all participants with normo-androgenic PCOS and without PCOS

	Total No. of Participants (***n*** = 255)		Women with NOR (***n*** = 145)		Women with normo-androgenic PCOS (***n*** = 110)		***Mann-Whitney test P***-value
Variables	Means ± SD	N (%)	Means ± SD	N (%)	Means ± SD	N (%)	
Age (years)	34.8 ± 1.4	–	34.6 ± 1.1	–	35.6 ± 1.2	–	0.31
BMI (kg/m^2^)	22.1 ± 1.5	–	21.3 ± 0.7		23.7 ± 1.0	–	0.12
No. of IVF attempts	2.3 ± 1.3	–	2.1 ± 1.5	–	2.2 ± 1.4	–	0.65
Age at menarche (years)	12.9 ± 1.1	–	12.8 ± 1.9	–	12.7 ± 1.7	–	0.17
**Infertility status**							
Primary infertility	–	136 (53.3)	–	88 (60.6)	–	48 (43.6)	–
Secondary Infertility	–	119 (46.6)		57 (39.3)	–	62 (56.3)	–
Duration of infertility (years)	3.6 ± 3.3	–	3.4 ± 1.1	–	3.5 ± 1.2	–	0.17
**Infertility diagnosis**							
Male factor	–	2 (0.78)	–	0	–	2 (1.8)	–
Female factor		146 (57.2)	–	48 (33.1)	–	98 (89)	–
Mixed factors	–	10 (3.92)	–	0	–	10 (9.0))	–
Unexplained factors	–	97 (38)	–	97 (66.8)	–	0	–
**Baseline serum hormonal evaluation on 2nd day of Menstrual cycle**							
FSH (IU/I)	6.5 ± 0.98	–	6.6 ± 0.42	–	5.87 ± 0.53	–	0.002
LH (IU/I)	6.8 ± 0.74	–	6.1 ± 0.55	–	8.3 ± 0.44	–	0.001
LH/FSH	1.02 ± 0.14	–	0.98 ± 0.07	–	1.84 ± 0.49	–	0.001
E2 (pg/ml))	44.01 ± 0.01	–	44.8 ± 1.13	–	43.2 ± 1.58	–	0.71
AMH (ng/ml)	5.8 ± 1.03	–	3.21 ± 1.14	–	8.13 ± 1.52	–	0.001
Prolactin (ng/ml)	15.6 ± 1.30	–	17.01 ± 1.17	–	14.77 ± 1.97	–	0.53
TSH (μIU/ml)	2.03 ± 0.98	–	2.17 ± 0.11	–	1.99 ± 0.17	–	0.18
Total T (nmol/L)	1.02 ± 0.89	–	0.98 ± 0.71	–	1.21 ± 0.19	–	0.05
Free T (nmol/L)	0.031 ± 0.11	–	0.02 ± 0.002	–	0.04 ± 0.002	–	0.35
SHBG (nmol/L)	57.49 ± 1.03	–	59.09 ± 1.21	–	55.74 ± 1.11	–	0.24
AFC	23.1 ± 1.63	–	15.94 ± 0.63	–	28.1 ± 0.48	–	0.001
**Stimulation protocol**		–		–		–	
Agonist	–	–	72 (49.6)	–	69 (62.7)	–	–
Antagonist	–	–	73 (50.3)	–	41 (37.2)	–	–
Total days of stimulation	10.2 ± 1.20	–	9.99 ± 1.4	–	10.1 ± 1.5	–	0.81
Total dose of gonadotrophins (IU/I)	2187 ± 198.74	–	2288.21 ± 101.32	–	1974.36 ± 106.3	–	0.001
rFSH	–	–	75 (51.72)	–	89 (80.9)	–	–
HP-hMG	–	–	70 (48.2)	–	21 (19.0)	–	–
**Serum endocrine level on hCG decision day**							
E2 (pg/ml))	1810.12 ± 111.89	–	1858.36 ± 211.89	–	1982.41 ± 332.13	–	0.52
P (ng/ml)	0.89 ± 0.31	–	0.85 ± 0.01	–	0.93 ± 0.02	–	0.14
LH (IU/I)	1.99 ± 0.44	–	1.98 ± 0.98	–	2.01 ± 1.02	–	0.74
Endometrium at oocyte retrieval (mm)			10.25 ± 1.02		10.42 ± 1.05		0.63

*BMI* body mass index, *IVF* in-vitro fertilization, *FSH* follicle-stimulating hormone, *LH* luteinizing hormone, *E2* estradiol, *AMH* anti-Mullerian hormone, *TSH* thyroid-stimulating hormone, *T* testosterone, *SHBG* sex hormone-binding globulin, *AFC* antral follicle count, *rFSH* recombinant follicle-stimulating hormone, *HP-hMG* highly purified human menopausal gonadotropin, *P* progesterone. The values are presented as mean ± SD. *P*-value *(Mann-Whitney test*) < 0.05 considered statistically significant

**Table 2:** Reproductive outcomes of all patients (*n* = 255) and of each group: women with normo-androgenic PCOS (*n* = 110) and without PCOS having normal ovarian reserve (NOR) (*n* = 145)

Reproductive outcomes	Total No. of Participants (***n*** = 255)	Women with NOR (***n*** = 145)	Women with normo-androgenic PCOS (***n*** = 110)	***Mann-Whitney test P***-value
**Ovarian outcome parameters**				
No. of Follicles aspirated [median (95% population limit)]	16.20 (11.2; 20.6)	13 (10.3; 18.2)	17 (15.4; 25.6)	0.03
No. of oocytes Retrieved [median (95% population limit)]	13.02 (10.32; 18.3)	11 (8.2; 13.5)	14.6 (11.3; 20.8)	0.001
No. of MII- oocytes [median (95% population limit)]	9.81 (8.01; 13.8)	9.4 (7.9; 12.3)	10.5 (8.7; 15.8)	0.04
Rate of mature oocytes (%) (0–100)	80	84	76	0.001
No. of Fertilized oocytes [median (95% population limit)]	9.10 (7.2; 11.98)	8.91 (6.4; 11.3)	9.86 (7.7; 13.54)	0.04
Fertilization rate (mean ± SD)	6.01 ± 1.68	4.92 ± 1.96	8.65 ± 1.33	0.001
**Embryological outcome parameters**				
No. of Normal Fertilized oocytes (2PN) [median (95% population limit)]	8.21 (6.01; 10.2)	8.41 (5.9; 10.6)	8.03 (6.7; 10.55)	0.09
No. of Early cleaved zygotes [median (95% population limit)]	8.00 (5.01; 8.51)	8.01 (5.2; 9.97)	7.99 (4.5; 9.69)	0.71
No. of blastomeres (6–8 cells) with regular symmetry at day 3 median (95% population limit)]	7.01 (4.89; 8.74)	7.22 (4.87; 8.14)	7.19 (4.96; 9.01)	0.66
No. of High-Quality day 3 embryos [median (95% population limit)]	2.00 (1.05; 3.01)	2.10 (1.98; 3.21)	2.20 (2.01; 2.99)	0.73
Total fragmentation rate (%)	15	13	18	0.001
Blastulation rate (%) at day 5 (0–100)	43	48	36	0.001
Expended blastocyst rate (%) at day 5 (0–100)	46	49	37	0.001
**Clinical outcomes**				
Clinical pregnancy rate per transfer (%) (0–100)	35	39	26	0.001

The values are presented as median (95% population limit) and percentage (%) point scale (0–100). *P*-value *(Mann-Whitney test*) < 0.05 considered statistically significant. Blastocyst rate was based on the number of blastocysts obtained / the number of zygotes cleaved. The number of fertilized oocytes means the total number of oocytes that were fertilized. A number of normally fertilized oocytes mean total fertilized oocytes obtained / abnormally fertilized oocytes. The fertilization rate is based on the number of oocytes obtained / the number of zygotes cleaved

### Altered miRNAs expression in human FF among PCOS and normal responders

**Fig. 1:**
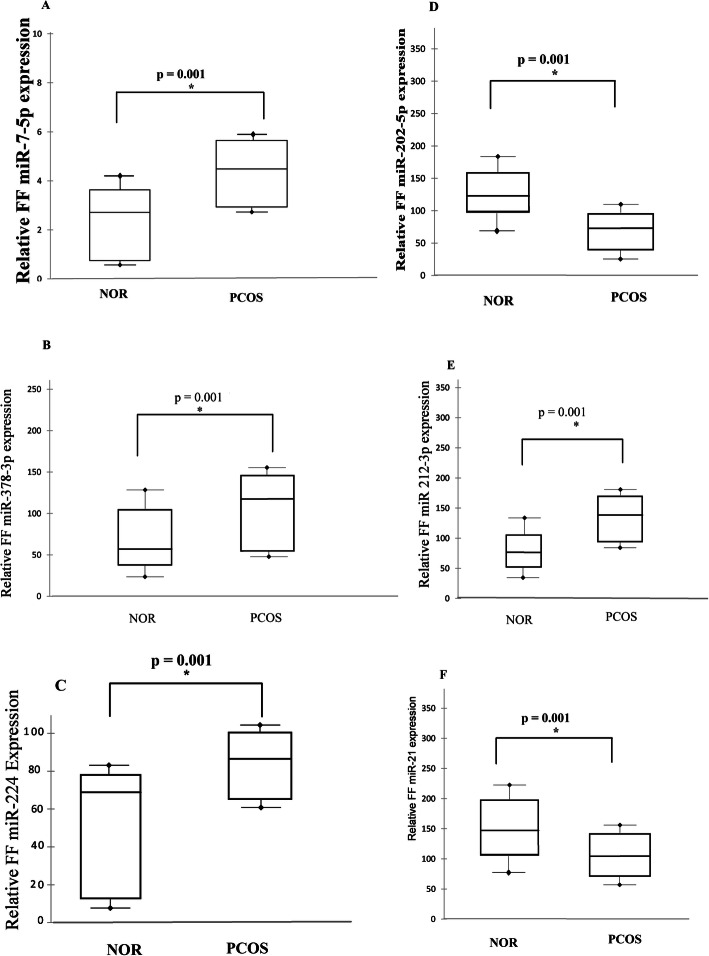
Relative miRNAs expression profile in follicular fluid (FF) pools of NOR patients (*n* = 145) compared to PCOS women (*n* = 110). **a** FF miR-7-5p **b** FF miR-378-3p **c** FF miR-224 **d** FF miR-202-5p **e** FF 212-3p **f** FF miR-21-5p. *P* < 0.05 is considered statistically significant (Mann-Whitney test)

Comparative expression analysis revealed significant lower abundance levels of miRNA-202-5p and miR-21-5p (*p* = 0.001), and higher abundance levels of miR-7-5p, miR-378-3p, miR-224, miR-212-3p (*p* = 0.001) in pooled samples of normo-androgenic PCOS (*n* = 110) compared to women without PCOS (*n* = 145) as shown in Fig. 1a-f. The fold change analysis of FF miRNAs at oocyte retrieval day among PCOS and NOR patients was listed in Table S1, showing no significant change in the fold change expression of miRNA-320a in the follicular fluid of both groups.

**Fig. 2:**
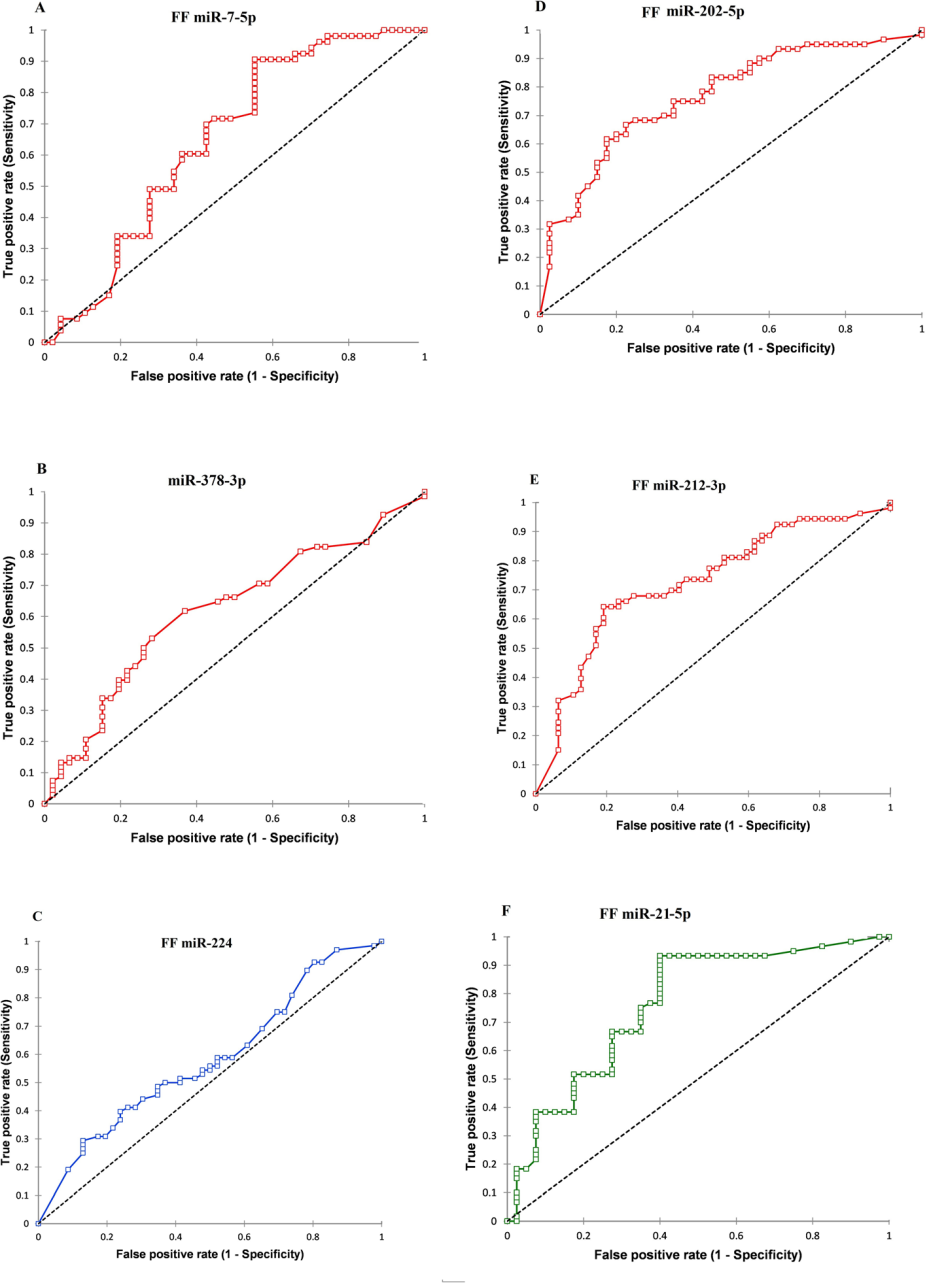
Receiver operating characteristic (ROC) analysis is used to find the power of discrimination of upregulated and downregulated FF miRNAs relative expression profile for PCOS diagnosis. **a** FF miR-7-5p **b** FF miR-378-3p **c** FF miR-224 **d** FF miR-202-5p **e** FF miR-212-3p **f** FF miR-21

The multivariate regression analysis exhibited that the six-miRNA signature was independent prognostic factor associated with PCOS after adjusting for body mass index (BMI) (adjusted OR 5.21 [1.79; 12.75], *p* = 0.001; 0.6 [0.27; 0.97], *p* = 0.001; 5.01 [1.75; 12.03], *p* = 0.001; 5.27 [1.89; 12.99], *p* = 0.005; 5.18 [1.99; 13.56]; *p* = 0.004; 0.98 [0.78; 0.99], *p* = 0.003 respectively as shown in Table 3. Receiver Operating Characteristics (ROC) curve analysis was used to evaluate the sensitivity, specificity, and area under the curve (AUC). The ROC plays a central role in determining the relationship between the differential expression of miRNAs (miR-7-5p, miR-202-5p, miR-378-3p, miR-224) and PCOS in the FF samples. For the discrimination of PCOS, the AUC values of the differential expression profile of miR-7-5p was (0.711 [0.63; 0.89], *p* = 0.001, cut-off point ≥0.931 with a sensitivity of 63.4% and a specificity of 87.1%; miR-378-3p, AUC 0.620 [0.51; 0.72], *p* = 0.001, cut-off point ≥25.1with a sensitivity of 68.9% and a specificity of 88.7; miR-224p, AUC 0.581 [0.47; 0.68], *p* = 0.001, cut-off point ≥0.987 with a sensitivity of 64.8% and a specificity of 86.4%; miR-202-5p, AUC 0.798 [0.59; 0.85], *p* = 0.003, cut-off point ≤15.8 with a sensitivity of 71.5% and specificity of 88.5%; miR-212-3p, AUC 0.726 [0.58; 0.89], *p* = 0.00, cut-off point ≥35.14 with a sensitivity of 78.8% and a specificity of 87.2%; miR-21-5p, AUC 0.643 [0.53; 0.76], *p* = 0.003, cut-off point ≤41.21 with a sensitivity of 61.5% and a specificity of 81.74% respectively (Table 4, Fig. 2a-f). However, the AUC value of miRNAs was increased to 0.881 [0.61; 0.92], *p* = 0.001 when combined in multivariate analysis. While the combination of these six miRNAs raised the sensitivity to 79.2% with a specificity of 87.32% (see Table 4).

**Table 3:** Multivariate logistic regression analysis exhibited the relationship between FF miRNAs relative expression with patients having polycystic ovaries

FF miRNAs relative expression related to normo-androgenic PCOS	Univariate logistic regression Analysis					Multivariate logistic regression Analysis				
***FF miRNAs***	***β***	***SE***	***Wald***	***Crude Odd ratio [95% Cl]***	***P-value***	***β***	***SE***	***Wald***	***BMI adjusted Odd ratio [95% Cl]***	***P-value***
miR-7-5p	0.81	0.038	141.36	5.01 [2.01; 11.02]	0.001	1.02	0.052	126.31	5.21 [1.79; 12.75]	0.001
miR-202-5p	0.97	0.041	132.7	0.74 [0.29; 0.89]	0.001	0.89	0.041	96.32	0.64 [0.27; 0.97]	0.001
miR-378-3p	0.89	0.032	48.62	4.93 [1.88; 10.36]	0.001	0.64	0.034	87.01	5.01 [1.75; 12.03]	0.001
miR-224	0.64	0.541	35.87	5.13 [1.91; 11.35]	0.004	0.53	0.059	96.32	5.27 [1.89; 12.99]	0.005
miR-212-3p	0.84	0.453	256.4	4.87 [1.68; 9.97]	0.003	0.99	0.034	52.14	5.18 [1.99; 13.56]	0.004
miR-21-5p	0.91	0.521	41.02	0.91 [0.89; 1.03]	0.002	1.23	0.061	75.97	0.98 [0.78; 0.99]	0.003

*FF* follicular fluid, *Cl* confidence interval, *β* beta coffecient, *SE* standard error

**Table 4:** Predictive values of sensitivity and specificity evaluation for the probability of PCOS

FF miRNAs	Prediction for normo-androgenic PCOS					
	AUC [(95% population limit)]	S.E.M	*P-Value	**Cut-off point	Sensitivity (%)	Specificity (%)
miR-7-5p	0.711 [0.63; 0.89]	0.061	**0.001**	≥ 0.931	63.4	87.1
miR-378-3p	0.620 [0.51; 0.72]	0.053	**0.001**	≥ 25.1	68.9	88.7
miR-224	0.581 [0.47; 0.68]	0.044	**0.001**	≥ 0.987	64.8	86.4
miR-202-5p	0.798 [0.59; 0.85]	0.054	**0.002**	≤ 15.8	71.5	88.5
miR-212-3p	0.726 [0.58; 0.89]	0.064	**0.001**	≥ 35.14	77.8	87.2
miR-21-5p	0.643 [0.53; 0.76]	0.057	**0.003**	≤ 41.21	61.5	81.74
Combination of FF miR-7-5p, 202-5p, 378-3p, 224, 212-3p, 21-5p	0.881 [0.61; 0.92]	0.040	**0.001**	–	79.2	87.32

*S. M.* E means ± standard error of the mean, *The null hypothesis was true area = 0.5, after adjusting a number of attempts and the number of embryos. *P*-values in bold letters are considered statistically significant *p* < 0.05. ** Estimated cut points that maximize sensitivity and specificity for observed range predictors

Our results revealed that expression of six miRNAs (miR-7-5p, miR-202-5p, miR-378-3p, miR-224, miR-21-5p, miR-212-3p) between patients with normo-androgenic PCOS and without PCOS give the significant AUC value with greater sensitivity and specificity together. Based on these findings, we proposed that these miRNAs signatures might be a potential noninvasive biomarker for the discrimination between normo-androgenic PCOS and non-PCOS patients.

### MiRNAs expression profile in follicular fluid (FF) samples in response to the exogenous gonadotropin therapy

**Fig. 3:**
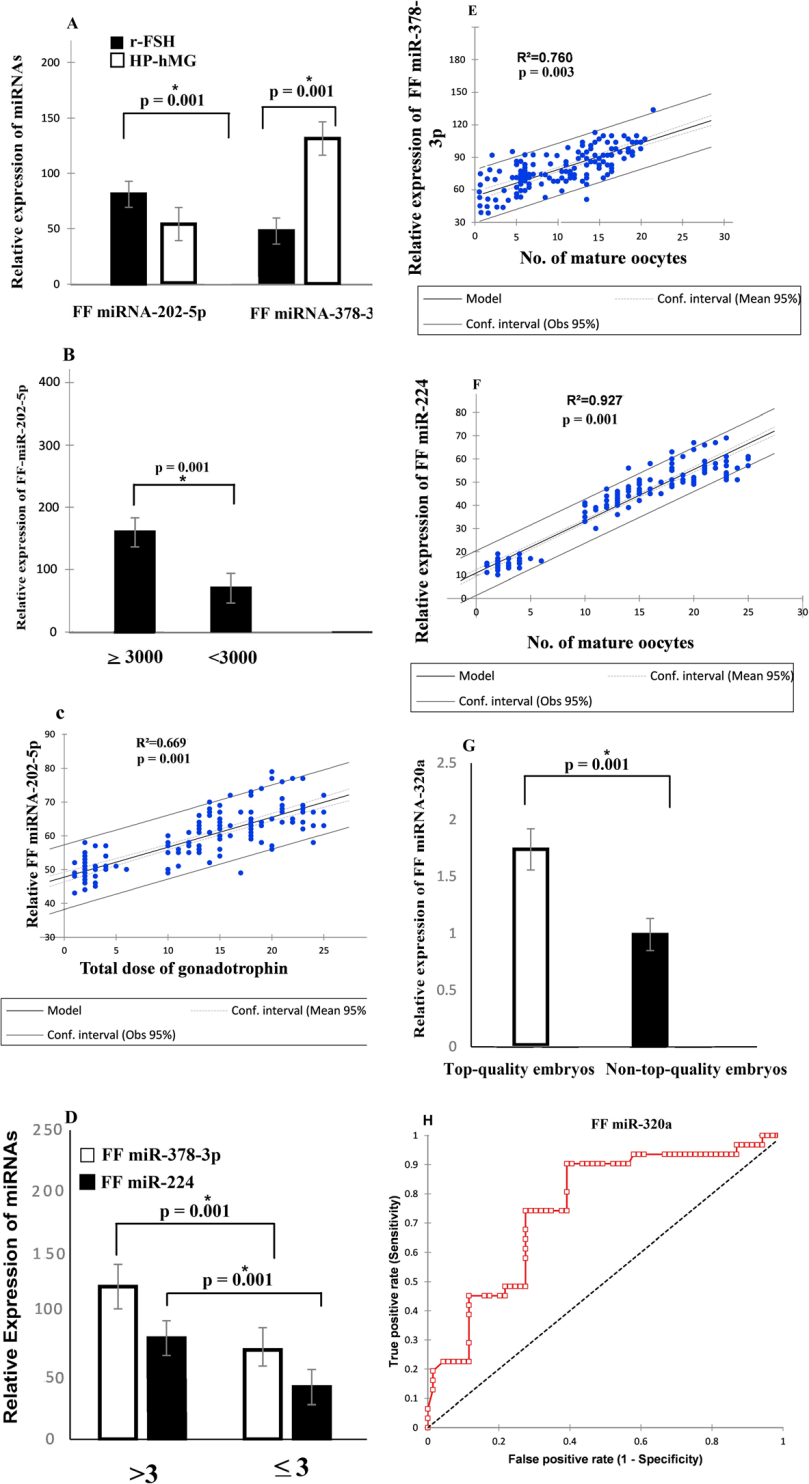
**a** Comparative expression profile of FF miR-202-5p and FF miR-378-3p in NOR patients relative to the gonadotropin type. rFSH (*n* = 80) (recombinant follicle-stimulating hormone), HP-hMG (*n* = 65) (highly purified human menopausal gonadotropin (*Mann-Whitney test, p* = 0.001). **b** Expression profile of FF miR-202-5p in NOR patients relative to the total dose of gonadotropin (≥ 3000, *n* = 90, ≤ 3000, *n* = 55) Mann*-Whitney test, p* = 0.001. **c** The relative expression of FF miR-202-5p has a significant but positive correlation with the overall dosage of gonadotropins (*R*^2^ = 0.669, *p* = 0.001). **d** Differential expression of FF miR-378-3p and FF miR-224 in NOR patients according to the number of retrieved oocytes on day 3 (≥ 3, *n* = 125, ≤ 3, *n* = 20) *Mann-Whitney test, p* = 0.001. **e** Spearman correlation coefficient depicted a significant positive association between FF miR-378-3p and the number of mature oocytes in NOR patients (*n* = 145). **f** The relative expression of FF miR-224 has a significant positive correlation with the number of mature oocytes in NOR patients. **g** Relative expression profile of FF miR-320a in NOR patients between top-quality embryos and non-top-quality embryos (*Mann-Whitney test, p* = 0.001). **h** The receiver operating characteristic (ROC) curve showing the discriminative capability of the FF miR-320a expression profile to predict embryo quality in NOR patients (*n* = 145)

The differential miRNA expression profile in FF samples was evaluated in response to the exogenous gonadotropin administration in NOR patients (*n* = 145). The miR-202-5p and miR-378-3p exhibiting modest expression differences according to the gonadotropin treatment. Remarkably, significantly lower abundance level of FF miR-202-5p and higher abundance levels of FF miR-378-3p was reported in pooled samples from patients treated with human menopausal gonadotropin (hMG) for those treated with recombinant follicle-stimulating hormone (rFSH) (*p* < 0.001) respectively, as presented in Fig. 3a. Furthermore, without concerning the type of treatment, the expression of miR-202-5p was significantly increased in FF pool samples from those patients with higher doses regimen of gonadotropin (≥ 3000 IU/l) than those who received < 3000 IU/I (*P* = 0.001), as displayed in Fig. 3b.

Similarly, the correlation coefficient exhibited that miR-202-5p has a significant but positive correlation with the overall dosage of gonadotropins (*R*^2^ = 0.669, *p* = 0.001) (see Fig. 3c). Patients with a low proportion of mature oocytes (MII) retrieved (≤ 3) showed a significantly lower concentration of FF miR-378-3p and miR-224 levels than those with more than 3 (*p* = 0.001) oocytes retrieved after 36 h post hCG administration (Fig. 3d). Spearman correlation coefficient showed that the relative expression of miR-378-3p and miR-224 levels was significantly associated with the number of mature oocytes (*R*^2^ = 0.760, *p* = 0.003, *R*^2^ = 0.927, *p* = 0.001), respectively (Fig. 3e-f).

### FF miR-320a and day 3 embryo quality

Our study expressed a significant association between FF miRNAs and day 3 high-quality embryos in NOR patients; we found that miRNA-320a was significantly different in top-quality embryos versus non-top-quality embryos on day 3 (*p* = 0.001) as depicted in Fig. 3g. Accordingly, miRNA-320a had an AUC of [0.753 (0.651; 0.855), *p* = 0.001], cutoff value ≥1.35 with a sensitivity of 80% and specificity of 71% respectively (Fig. 3h).

### FF miR-212-3p expression level and blastulation

**Fig. 4:**
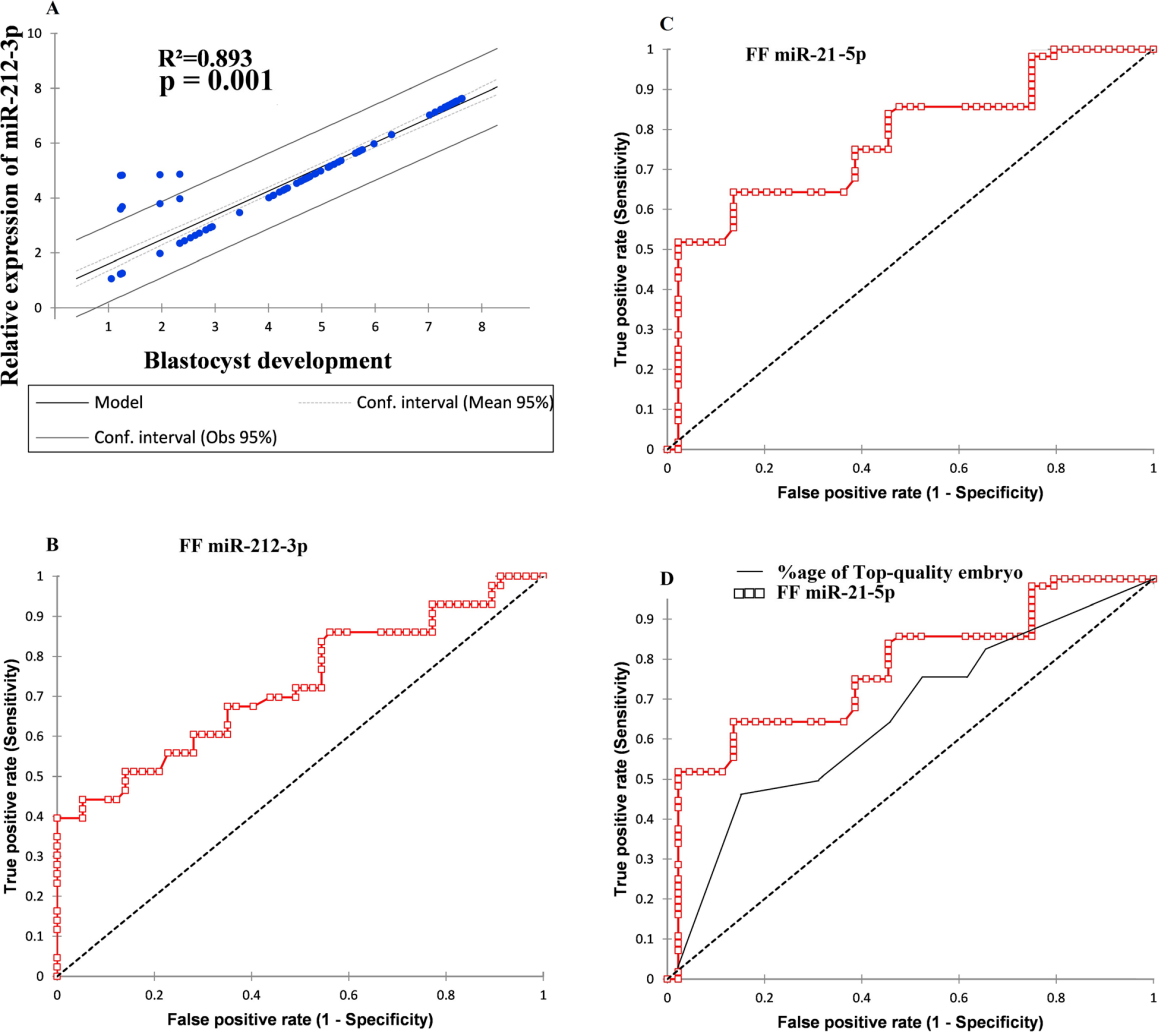
**a** The relative expression of FF miR-212-3p is significantly positively associated with blastocyst development in women with the normal ovarian reserve (*n* = 145). **b** Receiver operating characteristic (ROC) analysis revealed that FF miR-212-3p expression is a promising biomarker to predict blastocyst formation in NOR patients (*n* = 145). **c** The receiver operating characteristic (ROC) curve illustrated the discriminative ability of FF miR-21-5p to predict the clinical pregnancy outcome in NOR patients (*n* = 145). **d** Comparative ROC curve exhibiting discrimination between the relative expression profile of FF miR-21-5p and top-quality embryo proportion for clinical pregnancy outcome in NOR patients (*n* = 145)

We observed an a significant positive relationship between miR-212-3p expression level and high-quality blastocyst development (*R*^2^ = 0.893, *p* = 0.001) by analyzing the group of women without PCOS (*n* = 145) as shown in Fig. 4a. In fact, a high abundance of FF miR-212-3p was significantly associated with normo-androgenic PCOS. In our study, we noticed that the expression level of FF miR-212-3p was significantly related to the probability of embryos to develop into a high-quality blastocyst in patients with normal ovarian reserve (crude odds ratio = 1.32 [0.98; 2.01], *p* = 0.03 while the AUC value of FF miR-212-3p to predict blastulation was 0.744 (0.648; 0.841) with 79% sensitivity and 69% specificity at the cutoff of ≤20.1 (see Fig. 4b). Similarly, in NOR patients, the intrafollicular expression level of miR-212-3p was significantly correlated with the likelihood of achieving an expanded blastocyst (crude odds ratio = 1.31 [0.98; 2.01], *p* = 0.01 with an AUC value of 0.726 (0.623; 0.829 having 71% sensitivity and 88% specificity at the cutoff point ≤15.43).

### FF miR-21-5p expression level is associated with clinical pregnancy outcome

In NOR patients (*n* = 145), the level of FF miRNA-21 was associated with clinical pregnancy outcome [crude odds ratio = 1.99 [0.99; 3.97], *p* = 0.03 as given in Table S2. Furthermore, ROC curve analysis showed that the possible AUC value of FF miRNA-21 to predict clinical outcome was 0.774 (0.682; 0.865), *p* = 0.001 with a sensitivity of 74.8% and specificity of 83.7% at the cutoff point of ≥63.68 (Fig. 4c). Additionally, when we compared the discrimination power of FF miRNA-21 and the percentage of good quality embryos for predicting clinical pregnancy, we found that AUC value of about 0.78 (0.589; 8.712), *p* = 0.001, which was much greater than that for the percentage of good quality day 3 embryos (6–8 cells) (Fig. 4d).

### Predicted pathway analysis

**Fig. 5:**
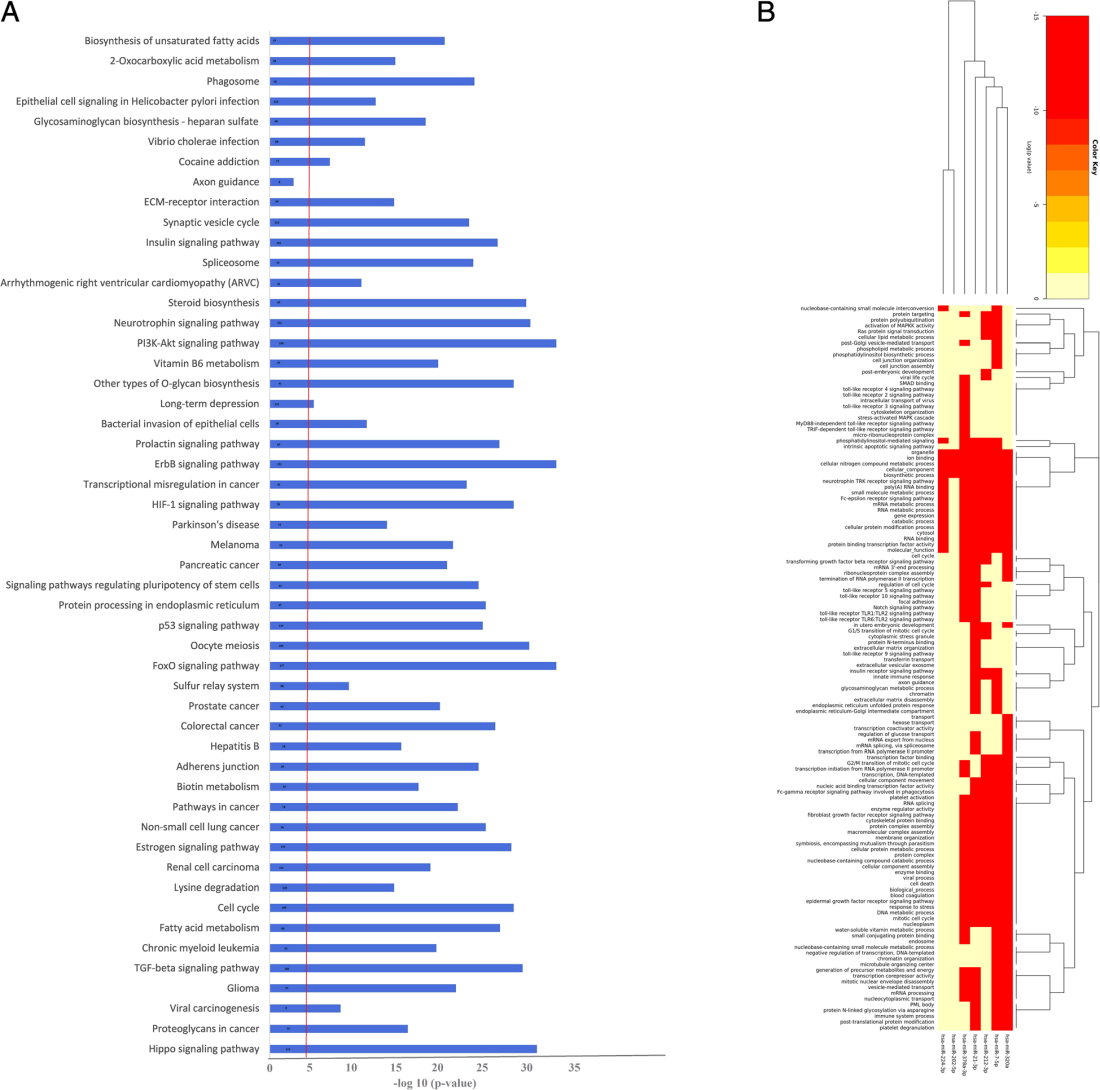
**a** Functional pathways Analysis- Top GO biological processes were identified using DAVID blue bars denoted pathways enriched among miRNAs detected in all FF samples of patients based on differential expression of studied miRNAs between normo-androgenic PCOS and NOR patients. Small numbers in each blue bar presented a number of gene count in each enriched term are associated with those miRNAs. The red line exhibited a statistically significant FDR thresh-hold. Benjamini and Hochberg multiple testing correlation were used to adjust the *p*-values (−log 10 FDR adjusted *p* values = < 0.05). **b** Predicted pathways; Heatmap analysis of miRNAs red color shows lower *p* values, and high expression, yellow indicates intermediate expression levels

MiRNAs were uploaded for KEGG pathway enrichment analysis and Gene Ontology (GO). The genes common in six out of eight programs (miRanda, TargetScan, DIANA-microT, miRDB, PicTar, miRWalk, RNA hybrid, target gene prediction at EMBLE) were selected to predict targets. We identified significant enriched biological processes based on differential expression of studied miRNAs between normo-androgenic PCOS and NOR patients; the 51 topmost enriched biological processes were shown in Fig. 5a. The most significant biological events involving the targets of our miRNAs were related to cell proliferation, regulation of cellular processes, as well as developmental processes. These biological processes were further validated by available literature data [10, 35, 36]. It was established that TGF beta signaling pathway, Hippo signaling pathway, p53 signaling pathway, cell cycle regulation, oocyte meiosis, HF-1 signaling pathway, FoxO signaling pathway, MAPKK signaling pathway, Notch signaling pathway, insulin receptor signaling pathway, and EGF signaling pathway were all actively involved in ovarian pathophysiology (Fig. 5b). Our results indicate that the differential expression of studied miRNAs in FF was potentially associated with cell growth, differentiation, and developmental processes during folliculogenesis and oocyte maturation.

## Discussion

**Fig. 6:**
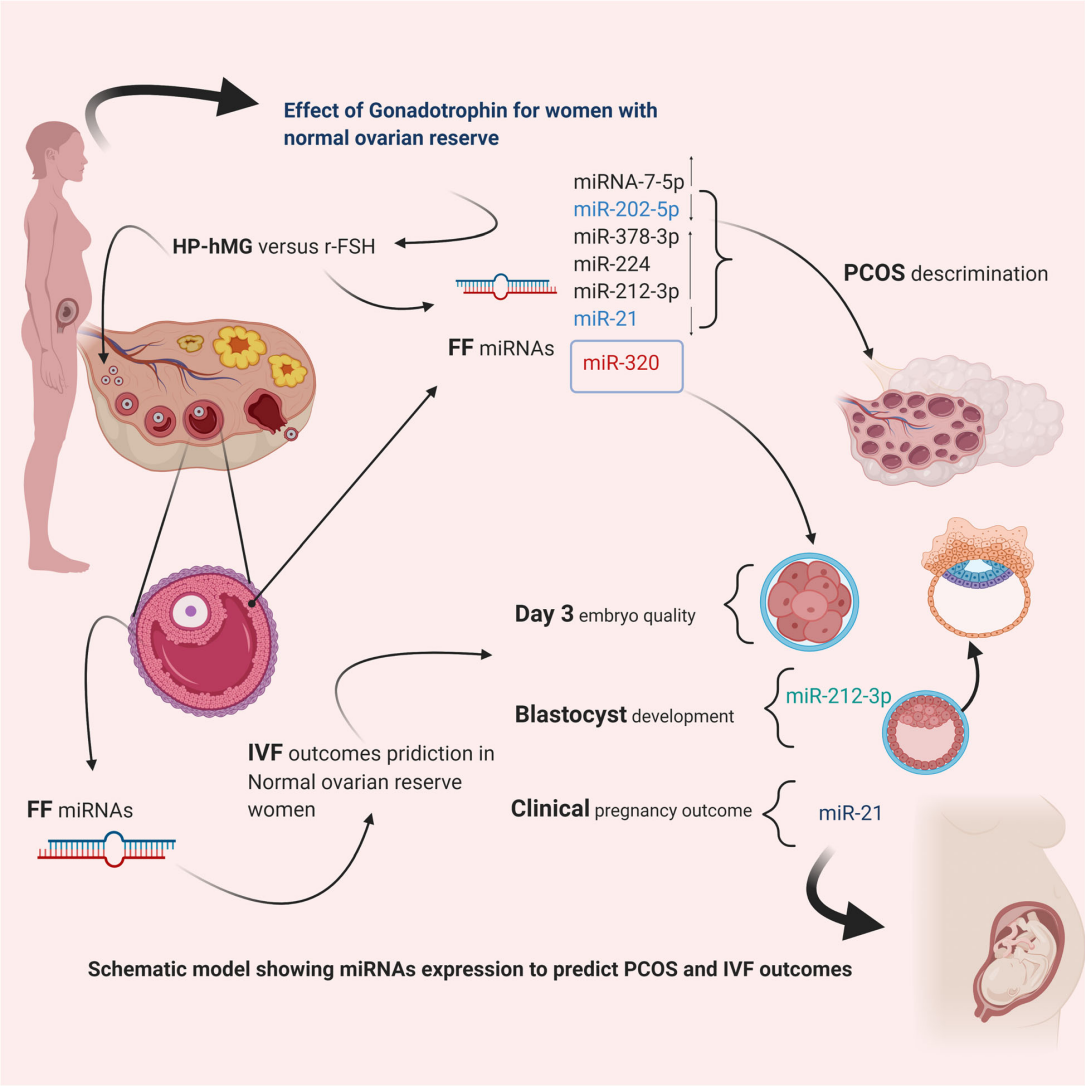
A schematic model showing miRNA expression profiles to predict PCOS and IVF outcomes in patients with normal ovarian reserve (NOR)

The present study explored seven circulating miRNAs (miR-7-5p, miR-202-5p, miR-378-3p, miR-224, miR-320a, miRNA-212-3p, and miR-21-5p) within the FF pool of normo-androgenic PCOS and NOR women undergoing treatment by IVF/ICSI. In particular, our study revealed that the expression levels of these miRNAs were significantly varied when compared to the patient’s ovarian reserve status, ovarian response to gonadotropin administration, and IVF outcomes (see Fig. 6).

Our data revealed that expression of miR-378-3p was significantly upregulated, and miR-202-5p was significantly lower abundance levels in FF pools from patients administered with human menopausal gonadotropin (hMG) in comparison with those treated with recombinant follicle-stimulating hormone (rFSH). This corresponds with the previous time course study conducted on a mouse model system, which demonstrated a biphase regulation of miRNAs through FSH in GCs. The study also observed that miR-202-5p and 14 other miRNAs were progressively downregulated by FSH exposure during the first 12 h but did not meet the significance level [37]. Inconsistent with our findings, the downregulation of miR-202-5p in GCs disturbs the oocyte-somatic cell interactions and severely interrupts the onset of primordial follicles’ development [38, 39]. It was subsequently noticed that downregulation of miR-202-5p results in reduced expression of one of the family members of KELCH, which has potential participation in the cross-talk between the oocyte and its companion somatic cell, disrupts the normal ovarian function exclusively in drosophila [39]. Furthermore, this hypothesis is strengthened by the SLIT-ROBO signaling pathway’s dysregulation due to reduced miR-202-5p levels in ovarian cancer, ultimately leading to an interruption in cellular communication across the ovarian tissues [38]. These findings clearly illustrate that the potential targets of miR-202-5p perform integrated regulatory functions and actively participate in follicular recruitment and growth. Recently, this was made clear by Gay et al. that knockout of the miR-202-5p gene in medaka ovaries reduces the expression of Gdf and Foxl2b genes that result in either no oogenesis or reduced oocyte production, which were later unable to mature and fertilize [24]. In mammals, FF miR-202-5p targeting the PI3K and TGFβR2 and positively associated with fertilization potential and oocytes maturation [40–43]. On these grounds, we suggest that the altered expression of miR-202-5p in human ovarian follicles might play a significant biological role in follicular development [40, 44].

PCOS is responsible for excessive follicle formation, signifying that the normal cross-talk between GCs and oocytes in mature preovulatory follicles might be badly mismanaged in the initial phase of development [45]. Our data of PCOS patients revealed an upregulation of miR-378-3p than in NOR patients. In mice ovaries, the upregulation of miR-378-3p leads to an increased density of primordial follicles and decreased apoptosis induction both in vivo and in vitro cultures [25]. Another study’s key finding suggested that the overexpression of miR-378-3p is responsible for an accelerated transition of follicles from primordial to the primary stages due to increased GC proliferation in anovulatory females [46]. Keeping all facts in mind, it is worth mentioning that abnormal preantral folliculogenesis due to overexpression of miR-378-3p might play a fundamental role in ovarian pathogenesis. It may be responsible for the relatively high density of the primary population of follicles in PCOS patients.

The significance of miRNAs in the early stages of embryonic development has been extensively discussed in several mammalian species [29]. Our results demonstrated that FF miR-320a was significantly linked to the embryonic developmental potential in NOR patients. Moreover, our findings showed that FF miR-320a was differentially expressed in top-quality embryos than impaired quality embryos. The recent publication using the mouse knockdown model for miR-320a revealed that the proportion of blastocyst-stage embryos was affected considerably by attenuating Wnt signaling components in early embryonic development both in vivo and in vitro [19, 47, 48]. Considering all these facts, it is inferred that miR-320 might be regarded as a potential contributor in regulating the embryo quality in patients undergoing IVF procedures [36].

Accumulative shreds of evidence indicate that miR-212-3p was preferentially enriched in FF and robustly expressed in preovulatory GCs and cumulus-oocyte complex (COC) of mammalian ovaries [10, 48, 49]. We found that FF miR-212-3p levels were significantly associated with embryo developmental potential in NOR patients. Therefore, the significant dysregulation of miR-212-3p expression in FF samples probably reflects the abnormal endocrine environment, impairing oocyte maturation. Comparably, the downregulation of miR-212-3p induces decreased implantation potential in cryopreserved vitrified mouse blastocysts due to the pervasive aberrant expression of a panel of genes necessary for embryo implantation [50]. Previous studies have shown that miR-212-3p also has a regulatory contribution to follicular development and steroidogenesis [51–53]. Specifically, experiments with the bovine model revealed that FF miR-212-3p may also play a decisive role in regulating FIGLA, an oocyte-specific transcriptional factor belonging to a basic family Helix-loop-Helix dimeric protein that is essential for regulating primordial follicles’ formation [54, 55]. In an in vitro study, FIGLA was known to bind promoter elements (E-Box) in the oocyte-zona-pellucida glycoprotein genes (Zp1, Zp2, Zp3), regulate their expression profile, and can effectively influence the blastocyst development [56]. Hence, the FF miR-212-3p expression evaluation could define the best embryo culture strategies for blastocyst development and IVF outcomes in patients with normal ovarian reserve.

Our study found higher abundance levels of FF miR-224 in PCOS patients than in NOR patients. Analysis of the previous literature has shown that overexpression of miR-224 of cumulus cells led to the cell expansion-dependent impairment and downregulation of gene expression associated with the oocyte maturation, most specifically by targeting Ptx3 [26]. Moreover, our results revealed that FF miR-224 level was significantly decreased in those NOR patients who have a small number of mature oocytes (≤ 3) than those with a more significant number of (> 3) oocytes retrieved (*p* = 0.001). In situ hybridization through locked-nucleic-acid-modified probes revealed that miR-224 is mainly expressed in GCs of follicles at varying degrees of development and suggestively increased the proliferation potential of GCs. Moreover, transfected GCs by miR-224 inhibitors halt their proliferation potential predominantly during follicle differentiation by altering the steroidogenic function of GCs [31].

As alluded to earlier, the phenomenon of embryo implantation is a crucial phase in early pregnancy establishment in mammals, but endometrium receptivity remains a limiting step in the overall success of IVF in humans. Our results showed that FF miR-21-5p differential expression significantly predicts the NOR group’s clinical pregnancy outcome with much higher sensitivity than the percentage of good quality embryos. Previous work demonstrated that FF miR-21-5p has a dynamic role in preimplantation embryo development by regulating apoptotic proteins [57, 58]. Moreover, downregulation of miR-21-5p in PCOS contributes to cell apoptosis by targeting PI3K/AKY and JAK/STAT3 signaling pathways [52]. It has been demonstrated experimentally that SMAD7 is a principal mediator and regulator of the TGF-beta signaling, which is one of the critical pathways involving in folliculogenesis, directly targeted, and inhibited by downregulation of miR-21-5p in PCOS [54].

Gene-GO terms demonstrated that the differential expression of miRNAs (up and down-regulation) might be responsible for the massive alterations in the harmony of the biological system. KEGG enrichment analysis highlights the presumptive targets of the studied miRNAs into major interactive signal transduction pathways. While pathway enrichment analysis revealed that the TGF-βeta signaling pathway, AKT pathway, and Hippo signaling pathway regulate normal ovarian function and folliculogenesis [17]. Mainly, one critical downstream mediator of the PI3K/AKT pathway, such as active FoxO family members, performs a crucial role during ovarian folliculogenesis by inhibiting primordial follicles’ activation. However, inactivation of FoxO3 leads to premature stimulation of primordial follicles and depletion of mature follicles [59]. A brief overview of the *in-silico* computational strategy demonstrated that FoxO3 is a promising target of miR-21-5p, which might be projected as an intricate network in PCOS, contributing through androgenic excess [35]. Notably, regulation of these miRNAs by various intrinsic and extrinsic factors in conjunction with their underlying mechanism of gene targeting generates complex networks of regulatory elements that have a substantial role in disease etiology, such as PCOS. One of this study’s strengths was the same age-matched large groups of NOR, and PCOS women, who were all undergoing IVF procedures with the attention of subsequent studied based on FF.

This study’s limitation is that the analysis was from different COS protocols that might affect miRNA expression. A more precise assessment of miRNA’s role requires using the same COS protocol in all patients. A more precise assessment of the role of miRNA in predicting individual embryo outcome would require the analysis of individual FF and tracking that through to the individual blastocyst stage.

## Conclusion

Conclusively, our results provide evidence that miR-7-5p, miR-378-3p, miR-224, miR-212-3p were a differentially high expression in normo-androgenic PCOS patients than NOR patients. While miR-202-5p and miR-21-5p were found significantly high levels in NOR patients versus PCOS patients. Similarly, the expression level of FF miR-212-3p was significantly related to the probability of embryos to develop into a high-quality blastocyst in patients with normal ovarian reserve. In the future, these miRNAs in FF could improve personalized IVF treatment strategies while treating infertility. More informatic considerations are required to highlight the detailed biological input of these miRNAs in the FF microenvironment to predict embryo quality, their response to gonadotropin therapy, and identifying their direct contribution to female infertility.

## Supplementary Information

**Additional file 1 : Table S1.** Fold change in the FF miRNAs at oocyte retravel day between women with normo-androgenic PCOS (*n* = 110) versus NOR women (*n* = 145). **Table S2.** Univariate analysis exhibiting association of specific FF miRNAs with blastocyst formation and pregnancy outcome.

## Data Availability

The data set used and analyzed during the current study is available from the corresponding author on reasonable request.

## Abbreviations

IVF: In vitro fertilization
AFC: Antral follicle count
AMH: Anti-Müllerian hormone
PCOS: Polycystic Ovarian Syndrome
NOR: Normal ovarian reserve
FF: Follicular fluid
miRNA: MicroRNA
ROC: Receiving operating characteristics
E2: 17β-estradiol
BMI: Body mass index
FSH: Follicular stimulating hormone
LH: Luteinizing hormone (LH)
TSH: Thyroid-stimulating hormone
TVS: Transvaginal ultrasonography
COS: Controlled ovarian stimulation
ICSI: Intracytoplasmic sperm injection
CI: Confidence interval
