# Supplementary material for: Extracellular microRNAs: key players to explore the outcomes of in vitro fertilization
Source: Reprod Biol Endocrinol. 2021 May 15;19:72. doi: 10.1186/s12958-021-00754-9 (PMC8122550; doi:10.1186/s12958-021-00754-9)
Supplement: Supplementary file 1 — Additional file 1 : Table S1. Fold change in the FF miRNAs at oocyte retravel day between women with normo-androgenic PCOS (n = 110) versus NOR women (n = 145). Table S2. Univariate analysis exhibiting association of specific FF miRNAs with blastocyst formation and pregnancy outcome. [file 12958_2021_754_MOESM1_ESM.docx]

**Table S1:** Fold change in the FF miRNAs at oocyte retravel day between women with normo-androgenic PCOS (n = 110) versus NOR women (n = 145)

| **PCOS vs. NOR** | **Fold Change** | **P-value** | **FDR** |
| --- | --- | --- | --- |
| miR-7-5p | 2.10421 | 0.0016 | 0.0015 |
| miR-378-3p | 1.97698 | 0.0029 | 0.0031 |
| miR-224p | 2.40674 | 0.0069 | 0.0058 |
| miR-202-5p | -1.96987 | 0.0011 | 0.0010 |
| miR-212-3p | 1.98397 | 0.0078 | 0.0041 |
| miR-21 | -2.04893 | 0.0024 | 0.0030 |
| miR-320a | 0.1365 | 1.032 | 0.0045 |

**Note:** P-value <0.05 considered statistically significant, and fold change values are in Log_2_ scale

**Table S2:** Univariate analysis exhibiting association of specific FF miRNAs with blastocyst formation and pregnancy outcome.

| **Relative FF microRNAs expression related to NOR** | **Blastocyst formation** |  | **Expended blastocyst formation** |  | **Clinical pregnancy outcome** |  |
| --- | --- | --- | --- | --- | --- | --- |
|  | ***Crude Odd ratio [95% Cl]*** | ***P-value*** | ***Crude Odd ratio [95% Cl]*** | ***P-value*** | ***Crude Odd ratio [95% Cl]*** | ***P-value*** |
| FF miR-212-3p | 1.32 [0.98; 2.01] | 0.03 | 1.31 [0.98; 2.01] | 0.01 | - | - |
| FF miR-21-5p | - | - | - | - | 1.99 [0.99; 3.97] | 0.03 |
